# Activity Recognition Using Community Data to Complement Small Amounts of Labeled Instances †

**DOI:** 10.3390/s16060877

**Published:** 2016-06-14

**Authors:** Enrique Garcia-Ceja, Ramon F. Brena

**Affiliations:** Tecnológico de Monterrey, Campus Monterrey, Av. Eugenio Garza Sada 2501 Sur, Monterrey, N.L. 64849, Mexico; ramon.brena@itesm.mx

**Keywords:** activity recognition, personalization, accelerometer

## Abstract

Human Activity Recognition (HAR) is an important part of ambient intelligence systems since it can provide user-context information, thus allowing a greater personalization of services. One of the problems with HAR systems is that the labeling process for the training data is costly, which has hindered its practical application. A common approach is to train a general model with the aggregated data from all users. The problem is that for a new target user, this model can perform poorly because it is biased towards the majority type of users and does not take into account the particular characteristics of the target user. To overcome this limitation, a user-dependent model can be trained with data only from the target user that will be optimal for this particular user; however, this requires a considerable amount of labeled data, which is cumbersome to obtain. In this work, we propose a method to build a personalized model for a given target user that does not require large amounts of labeled data. Our method uses data already labeled by a community of users to complement the scarce labeled data of the target user. Our results showed that the personalized model outperformed the general and the user-dependent models when labeled data is scarce.

## 1. Introduction

In recent years Human Activity Recognition (HAR) [1,2] has gained a lot of attention because of its wide range of applications in several areas, such as health and elder care, sports, *etc.* [3,4,5]. Inferring the current activity being performed by an individual or group of people can provide valuable information in the process of understanding the context and situation of a user in a given environment, and as a consequence, personalized services can be delivered. Recently, the use of wearable sensors has become the most common approach to recognize physical activities because of its unobtrusiveness and ubiquity—specifically the use of accelerometers [5,6,7,8] because they are already embedded in several devices and they raise less privacy concerns than other types of sensors.

One of the problems in HAR systems is that the labeling process for the training data tends to be tedious, time consuming, difficult, and prone to errors. This problem has really hindered the practical application of HAR systems, limiting them to the most basic activities for which a general model is enough, as is the case for the pedometer function or alerting the user who spends too much time sitting down; both functions are now available in some fitness devices and smartwatches.

On the other hand, when trying to offer personalized HAR systems, there is the problem that at the initial state of the system there is little or no information at all (in our case, sensor data and labels). In the field of *recommender systems* (e.g., movie, music, book recommenders), this is known as the *cold-start problem* [9] and it includes the situation when there is a new user but nothing or little is known about him/her, in which case it becomes difficult to recommend an item, service, *etc*. It also encompasses the situation when a new item is added to the system but—because no one has yet rated, purchased, or used that item—it is difficult to recommend to anybody.

In this work, we will focus on the situation when there is a new user in the system and we want to infer her/his physical activities from sensor data with high accuracy, even when there is little information about that particular user—assuming that the system already has data from a community of users and also that their associated data is already labeled. We are thus using a “crowdsourcing” approach that consists of using collective data to fit personal data. The key insight in our approach is that instead of building a model with all the data from all other users, we will use the scarce labeled data from the target user to select a subset of the other users’ data based on class similarities in order to build a personalized model. The personalization of a model is relevant because the kinematics between individuals vary, so we want to exclude instances from the training set that are very different from those of the target user in order to reduce noise. In our previous work [10], we proposed a method to select meaningful instances from the community data by comparing them to the scarce labeled instances of the target user based on class similarities. In this work, we extend this idea to the case when there are no labeled data of the target user, which we will refer to as the *zero-labeling* case. We will use a self-learning approach to automatically label a portion of the data and use it as ground truth to build the personalized model.

This paper is organized as follows: Section 2 presents related work about HAR systems. Section 3 details the process of building a Personalized Model for the case of scarce and *zero labeled* data. Section 4 introduces the public datasets that were used for the experiments. The experiments are described in Section 5. Finally in Section 6 we draw our conclusions.

## 2. Related Work

Being able to infer the activities currently performed by the user is of great interest because of the wide range of possible applications of it; so, in the last years there have been many works in this area. Activity recognition can be very useful in medicine, Ambient Intelligence (AmI) [11], indoor location [12], *etc*. Knowing the current activity being performed by the user can provide valuable information in the process of understanding the context and situation in a given environment.

There are two main types of sensors that have been used for Human Activity Recognition: *external sensors* and *wearable sensors*. External sensors are installed in the environment and may not have direct physical contact with the user. Examples of such sensors are: video cameras, microphones, motion sensors, depth cameras like the Microsoft Kinect, RFID tags, switches, *etc*. On the other hand, wearable sensors [2] are carried by the user or are embedded in devices such as smartphones, smartwatches and fitness bracelets. Examples of wearable sensors are: accelerometers, gyroscopes, magnetometers, Wi-Fi, Bluetooth, *etc.* (Wi-Fi and Bluetooth are of course communication protocols, but the Wi-Fi and Bluetooth sensing capabilities of devices like smartphones can be used, for instance, for indoor location or proximity detection.)

The main *external sensors* that have been used for activity recognition are video cameras and sensors installed in smart environments. Regarding the use of video cameras, Bobick *et al.* [13] proposed a method based on temporal templates which first constructs a motion energy image (MEI) and then a motion history image (MHI) is generated. They evaluated their method using aerobics data consisting of 18 exercises. Roberton *et al.* [14] proposed a hierarchical general method for high-level behavior recognition.

Activities that involve interactions between persons (e.g., shake hands, hug, *etc*.) have also been studied [15]. In recent years, the use of the Microsoft Kinect has gained a lot of attention. One of the advantages of this sensor is that it also captures information about the depth of each of the points which makes it more robust in changing light conditions. Sung [16] used this sensor to recognize 12 different activities performed by four people.

A smart environment is a physical place with several installed sensors. It can be a single room, a house, or an entire building. In a smart environment, all the information gathered by all these sensors is used to understand the context of that environment in order to provide assistance, recommendations, and services to the inhabitants. In the work of Kasteren *et al.* [17], a sensor network setup that can be easily installed and used in different houses is presented. In their experiments, they used 14 state change sensors located in doors, cupboards, a refrigerator, and a toilet flush sensor. Some of the activities they recognized were showering, sleeping, breakfast, dinner, *etc*. In the work of Klack *et al.* [18], an intelligent floor was installed in a smart home for an elderly and health care application. This floor is intended to detect the user’s position, falls, and abnormal patterns. Amoretti reports [19] an activity recognition system which takes into account the user position, posture, gesture, and interactions through the use of cameras and environmental sensors.

On the other hand, the use of *wearable sensors* [2] has gained a lot of attention because they have several advantages; for example, the recognition can be performed anywhere, unlike video cameras in which it is restricted to a specific area. Another advantage is that wearable sensors like accelerometers and gyroscopes raise less privacy concerns compared to video cameras or microphones. Furthermore, in environments with multiple residents with external sensors it becomes difficult to detect which person activated a specific sensor. This is not a problem for wearable sensors, since they are personal. Given these advantages, this work uses data gathered from wearable sensors.

A common recent trend is to use smartphones, since they are provided with several sensors and can be easily programmed. Another advantage is that all the processing can be made inside the phone so there is no need to carry another processing unit. One of the first works to perform all the recognition inside a phone was the one of Brezmes *et al.* [20]. Other works that made use of smartphones were [5,21,22,23,24].

### 2.1. Types of Models

From the reviewed literature, three broad model categories in HAR can be identified—namely: *General*, *User-Dependent*, and *Mixed* models.

- *General Models (GM)*: Sometimes also called *User-Independent Models*, *Impersonal Models*, *etc.* From now on we will refer to them as GMs. For each specific user *i*, a model is constructed using the data from all other users *j*, j≠i; the accuracy is calculated testing the model with the data from user *i*.
- *User-Dependent Models (UDM)*: They are also called *User-Specific Models*; here we will refer to them as UDMs. In this case, individual models are trained and evaluated for a user using just her/his own data.
- *Mixed Models (MM)*: This type of model tries to combine GMs and UDMs in the hope of adding their respective strengths, and is usually trained using all the aggregated data without distinguishing between users. Lockhart & Weiss [25] call them “Hybrid models”.

There are some works in HAR that have used the UDM and/or GM approach [26,27,28]. The disadvantages of GMs are mostly related to their lack of precision, because the data from many dissimilar users is just aggregated. This limits the GM HAR systems to very simple applications, such as pedometers and the detection of long periods of sitting down.

The disadvantages of UDM HAR systems are related to the difficulties of labeling the specific users’ data, as the training process easily becomes time consuming and expensive, so in practice users avoid it. For UDMs, several techniques have been used to help users label the data, as it is the weakest link in the process. For example, Lara *et al.* [29] presented a mobile application in which the user can select several activities from a predefined list. In the work of Anguita *et al.* [30], they video-recorded the data collection session and then manually labeled the data. Some other works have used a Bluetooth headset combined with speech recognition software to perform the annotations [31] or manually annotate data from taking notes [32]. In any case, labeling personal activities remains very time-consuming and undesirable.

From the previous comments, apparently MMs look like a very promising approach, because they could cope with the disadvantages of both GM and UDM. However, in practice, combining the strengths of both has been an elusive goal. As noted by Lockhart & Weiss [25], no such system has made it to actual deployment. There have been several works that have studied the problem of scarce labeled data in HAR systems [33,34] and used semi-supervised learning methods to deal with the problem; however, they follow a *Mixed* model approach—*i.e.*, they do not distinguish between users.

### 2.2. Crowdsourcing and Model Personalization

Recently, the use of *crowdsourcing* approaches [35] has been explored for application in Human Activity Recognition systems. Leveraging the massive user participation made possible by Web 2.0 [36], the idea of *crowdsourcing* is to collect data from volunteers connected through the internet to a given site. Crowdsourcing has proven to achieve very valuable results for distributing tasks to the myriad anonymous users who solve many easy or some hard problems, such as molecule sequencing. The most paradigmatic example is Wikipedia, but in cases like the ones we are considering in our study, users participate mainly by allowing their connected devices to send data to a collecting site. In our case, we expect to collect a sizeable collection of tagged accelerometer data coming from a very large community of users (e.g., the nearly 10 million active users of the Fitbit devices [37])—with that community size, even a very small percentage of labeling (say an average of only one label reported per user) is enough to obtain a very large data bank (10 million data points).

The combination of the work of several non-expert workers has proven to outperform the work done by single expert sources. For example, in the work of Hahn *et al.* [38], they crowdsourced the task of synthesizing information and obtained results comparable to those of top information sources on the web. The use of crowdsourcing approaches for activity recognition is not new. For example, in the work of Kirkham *et al.* [39], they leveraged the error-prone task of defining activity annotation boundaries to a set of annotators to reduce “label-jittering” (activity start and end times do not align perfectly with the annotation). In the work of Heilbron and Niebles [40], the Amazon Mechanical Turk was used to recruit workers to annotate activities from video, and they achieved high quality annotations when combining the work of six annotators. Lasecki *et al.* [41] also used Mechanical Turk workers to annotate dependencies between actions to identify high-level home activities.

With the advent of new devices that have several embedded sensors—such as smartphones and smart-watches—it becomes possible to collect large quantities of data. The term Mobile Crowd Sensing has been used to refer to this scenario with a formal definition presented by Guo *et al.* [42] as: *“a new sensing paradigm that empowers ordinary citizens to contribute data sensed or generated from their mobile devices, aggregates and fuses the data in the cloud for crowd intelligence extraction and people-centric service delivery.”* One of the important areas that has benefited from Mobile Crowd Sensing is healthcare. For example, for the monitoring of tinnitus (the perception of noise in the ears) [43], asthma management [44], and mood recognition [45], to name a few. Other applications of crowd sensing are social network inference [46], traffic congestion avoidance [47], indoor location [48], *etc*. Currently, there is a project under development called Crowdsignals that aims to collect activity information using smartphone and smart-watch data [49].

Another important aspect of activity recognition is model personalization. Model *personalization/adaptation* refers to training and adapting classifiers for a specific user according to her/his own needs. Building a model with data from many users and using it to classify activities for a target user will introduce noise due to the diversity between users. Lane *et al.* [50] showed that there is a significant difference for the *walking* activity between two different groups of people (20–40 and >65 years old). Parviainen *et al.* [51] also argued that a single general model for activity classification will not perform well due to individual differences and proposed an algorithm to adapt the classification for each individual by only requesting binary feedback from the user. Lu *et al.* [52] used a model adaptation algorithm (Maximum A Posteriori) for stress detection using audio data. Zheng *et al.* [53] used a collaborative filtering approach to provide targeted recommendations about places and activities of interest based on GPS traces and annotations. They manually extracted the activities from text annotations, whereas in this work the aim is to detect *physical* activities from accelerometer data. Abdallah *et al.* [54] proposed an incremental and active learning approach for activity recognition to adapt a classification model as new sensory data arrives. Vo *et al.* [55] proposed a personalization algorithm that uses clustering and a Support Vector Machine that first trains a model using data from user A and then personalizes it for another person B; however, they did not specify how user A should be chosen. This can be seen as a 1 → n relationship in the sense that the base model is built using data from a specific user A and the personalization of all other users is based solely on A. The drawback of this approach is that user A could be very different from all other users, which could lead to poor final models. Our work differs from this in that we follow a n → 1 approach, which is more desirable in real world scenarios—*i.e.*, use data already labeled by the community users to personalize a model for a specific user. Lane *et al.* [50] also personalize models for each user by first building Community Similarity Networks (CSN) for different dimensions, such as: physical similarity, lifestyle similarity, and sensor-data similarity. Our study differs from this in two key aspects: First, instead of looking for inter-user similarities, we find similarities between classes of activities. This is because two users may be similar overall, but there may still be activities that are performed very differently between them. Second, we use only accelerometer data to find similarities, since other types of data (age, location, height, *etc.*) are usually not available or impose privacy concerns. Furthermore, we evaluated the proposed method on five different public datasets collected by independent researchers.

In this work, we will use an approach that is between GMs and UDMs, so it could be seen as a variation of Mixed Models. However, here we use a small amount of the user’s available data to select a subset of the other users’ activities instances to complement the data from the considered user, instead of just blindly aggregating all other users’ data. This selection is made based on class similarities and the details will be presented in Section 3. We also present some results for the case of “zero-labeling” that does not use any labeled data from the target user.

## 3. Personalized Models

In this section, we describe how a Personalized Model (PM) is trained for a given target user $u_{t}$. A General Model (GM) includes all instances from users $U_{other}$, where $U_{other}$ is the set of all users excluding the target user $u_{t}$. In this case, there may be differences between users in how they perform each activity (e.g., some people tend to walk faster than others). As a result, this approach will introduce noisy instances to the train set, and thus the resulting model will not be very accurate when recognizing activities for $u_{t}$.

The idea of building a PM is to use the scarce labeled data of $u_{t}$ to select instances from a set of users $U_{similar}$, where $U_{similar}$ is the set of users similar to $u_{t}$ according to some similarity criteria. Building PMs for activity recognition was already studied by Lane *et al.* [50], with the limitations we already explained in the preceding section. In our approach, we look for similarities on a per class instead of a per user basis—*i.e.*, the final model will be built using only the instances that are similar to those of $u_{t}$ for each class. Procedure 1 presents the proposed algorithm to build a PM based on class similarities.

The procedure starts by iterating through each possible class *c*. Within each iteration, instances of class *c* from the $u_{t}$ train set $\tau_{t}$ and all the instances of class *c* that belong to all other users are stored in $data_{all}$. The function subset(set,c) returns all instances in set of class *c* which are then saved in $data_{t}$. Function instances(U) returns all the instances that belong to the set of users *U*. Next, all instances in $data_{all}$ are clustered using the *k*-means algorithm for k=2,...,UpperBound. For each *k*, the *Silhouette* clustering quality index [56] of the resulting groups is computed and the *k* that produces the optimal quality index is chosen. A clustering quality index [57] is a measure of the quality of the resulting clustering based on compactness and separation. The *Silhouette* index was chosen because it has been shown to produce good results with different datasets [57]. Next, instances from the cluster in which the majority of instances from $data_{t}$ ended up are added to the final training set T. In addition, all instances from $data_{t}$ that ended up in other clusters are added to T to make sure all the data from $u_{t}$ are used. After the for loop, all instances in T are assigned an *importance* weight as a function of the size of $\tau_{t}$ such that instances from the $u_{t}$ train set have more impact, as more training data is available for that specific user. The exponential decay function $y={\left(1-r\right)}^{x}$ was used to assign the weights where *r* is a decay rate parameter and $x=\left|\tau_{t}\right|$. The weight of all instances in T that are not in $\tau_{t}$ is set to *y*, and the weight of all instances in $\tau_{t}$ is set to 1−y. Finally, the model is built using T with the new instances’ weights. Note that the classification model needs to have support for instance weighting. For the experiments, we used a decision tree implementation called rpart [58], which supports instance weighting.

**Procedure 1** Build PM1:$T\leftarrow \left\{\right\}$▷ Start with an empty train set2:**for**
*c* in *C*
**do**▷ For each class3:     $data_{t}\leftarrow subset\left(\tau_{t},c\right)$▷ $\tau_{t}$ is the target user’s train set4:     $data_{other}\leftarrow subset\left(instances\left(U_{other}\right),c\right)$5:     $data_{all}\leftarrow data_{t}\cup data_{other}$6:     Cluster $data_{all}$ using k-means for k=2,…,UpperBound and select the optimal *k* according to some clustering quality index.7:     S←${arg max}_{g\in G}\left|data_{t}\cap g\right|$▷ *G* is the set of the resulting *k* groups8:     $T\leftarrow T\cup S\cup data_{t}$9:**end**
**for**10:weight(T)    ▷ Assign a weight to each instance such that the importance of $\tau_{t}$ increases as more training data of the target user is available.11:Build model using training set T.

### Zero-Labeling Case

We call *zero-labeling* the case in which we do not have labeled data at all from the user under consideration, though we do have a wealth of labeled data from the community. This situation is important in practice, because we do not want a new device user to be required to label data (which is indeed a cumbersome and dull activity) in order to start using the activity recognition device in a useful way. Our premise is that—with a given database of already-labeled data from the community—by finding similarities in the data of this user with data from other users, it would be possible to perform high-quality activity recognition similar to the one when there is scarce labeling.

For the *zero-labeling* case, we adopted a self-learning approach, which is a type of Semi-supervised learning (SSL) [59]. Semi-supervised learning is between Supervised and Unsupervised learning. Let $X_{u}$ be the set of unlabeled instances and $X_{l}$ the set of labeled instances. One of the ideas of SSL is to use both $X_{l}$ and $X_{u}$ to generate a predictive model. One of the first introduced SSL methods was *self-learning*, which consists of training a model *M* using $X_{l}$. Then, a subset of $X_{u}$ instances are classified using *M* and used to retrain *M* [60].

We will use self-learning to infer the labels of some of the instances and then use those inferred labels as the target user’s training set. Let $X_{u}$ be the set of unlabeled instances of the target user. For the *zero-labeling* case, $X_{u}$ consists of all the target user’s data, since all instances are unlabeled. Then, use the model *M* trained with the data from all other users to predict the labels of a random subset *R* of $X_{u}$. The newly-predicted instances are used as ground truth—*i.e.*, add them to the train set $\tau_{t}$ and build the Personalized Model as usual (Procedure 1).

## 4. Datasets

We conducted our experiments with five publicly available datasets from the UCI Machine Learning repository [61]. The criteria for selecting the datasets were:

1. The dataset must include simple activities.
2. It must contain data collected from several users.
3. The information of which user generated each instance must be included.
4. Each class should have several instances per user.

Now we describe the details about each of the datasets that met the criteria to be considered in our experiments. We also include information about the processing steps we made for each of the datasets. Datasets vary in the number of users, classes, sensors, *etc*. The dataset with the greatest number of users that was found was D3 Wireless Sensor Data Mining (WISDM) with a total of 36 users. The biggest dataset in terms of number of instances was D1 Chest Sensor.

- *D1: Chest Sensor Dataset.* This dataset has data from a wearable accelerometer mounted on the chest [62,63]. The data were collected from 15 participants performing seven different activities. The sampling rate was set at 52 Hz. The sensor returns values for the *x*, *y*, and *z* axes. The included activities are: (1) working at computer; (2) standing up, walking, and going up/down stairs; (3) standing; (4) walking; (5) going up/down stairs; (6) walking and talking with someone; (7) talking.
  Since our focus is on simple activities, we discarded activities 2, 5, and 6 as they involve the performance of different actions in an interleaved or concurrent manner but with the same label (we will leave model personalization for more complex activities as future work, e.g., shopping, office work, cooking, *etc*.). To reduce signal noise, a moving average filter with a window length of 10 was applied to the raw accelerometer data for each axis. Then, we extracted 16 common statistical features on fixed length windows of size 208, which corresponds to 4 s. The features were: mean for each axis, standard deviation for each axis, maximum value of each axis, correlation between each pair of axes, mean of the magnitude, standard deviation of the magnitude, mean difference of the magnitude, and area under the curve of the magnitude. The features were ranked with a filter method based on information gain [64,65], and the top 10 were kept. The resulting total number of instances was 8506.
- *D2: Wrist Sensor Dataset.* This dataset is composed of the recordings of 14 simple activities performed by a total of 16 volunteers with a tri-axial accelerometer mounted on the right wrist [66,67]. The set of activities includes: (1) brush teeth; (2) climb stairs; (3) comb hair; (4) descend stairs; (5) drink glass; (6) eat meat; (7) eat soup; (8) get out of bed; (9) lie-down in bed; (10) pour water; (11) sit-down in chair; (12) stand-up from chair; (13) use telephone; and (14) walk. Activities 6 and 7 were excluded since there is only data from one user. The sampling rate was set at 32 Hz. The same pre-processing steps and the same set of features as dataset 1 were extracted from a window of size 128 that corresponds to 4 s. This resulted in a total of 2807 instances.
- *D3: WISDM Dataset.* This dataset was collected by 36 subjects while performing six different activities [21]. The data was recorded using a smartphone with a sampling rate of 20 Hz. The dataset already contained 46 features extracted from fixed-length windows of 10 s each. The activities include: (1) walking downstairs; (2) jogging; (3) sitting; (4) standing; (5) walking upstairs; and (6) walking. The total number of instances is 5418.
- *D4: Smartphone Dataset.* This database was built from the recordings of 30 subjects performing activities of daily living while carrying a waist-mounted smartphone with embedded inertial sensors [30,68]. The activities in this database include: (1) walking; (2) walking upstairs; (3) walking downstairs; (4) sitting; (5) standing; and (6) laying down. The sampling rate was set at 50 Hz. For our experiments, we used a subset of this dataset that was distributed in the “Data analysis” course [69] which consists of 21 users. The dataset already includes 561 extracted features from the accelerometer and gyroscope sensors. The total number of instances for the 21 users is 7352.
- *D5: Opportunity Dataset.* This dataset consists of daily activities recorded with body-worn sensors, object sensors, and ambient sensors [70,71]. We considered the four low-level activities: (1) stand; (2) walk; (3) sit; and (4) lie. We used the accelerometer data from the back and right-shoe inertial sensors, which was sampled at 30 Hz. The same pre-processing steps and the same set of features as dataset 1 were extracted for both sensors with a window of size 120 that corresponds to 4 s. The total number of instances for the four users is 1855.

For all the datasets, the features were normalized between 0 and 1. Table 1 shows a summary of the datasets and their characteristics.

## 5. Experiments and Results

Several works in HAR perform the experiments by first collecting data from one or several users and then evaluating their methods using *k-fold cross validation* (ten being the most typical value for *k*) on the aggregated data. For a k=10, this means that all data is randomly divided into 10 subsets of approximately equal size. Then, 10 iterations are performed. In each iteration, a subset is chosen as the test set and the remaining k−1 subsets are used as the train set. This means that 90% of the data is completely labeled and the remaining 10% is unknown; however, in real life situations, it is more likely that only a fraction of the data will be labeled. In our experiments, we want to consider the situation when the target user has only a small amount of labeled data. To resemble this, our models’ evaluation procedure consists of sampling a small percent *p* of instances from the target user $u_{t}$ to be used as the train set $\tau_{t}$ and uses the remaining data to test the performance of the General Model, User-Dependent Model, and our proposed Personalized Model. To reduce sampling variability of the train set, we used proportionate allocation stratified sampling. We chose *p* to range between 1% and 30% with increments of 1. For each *p* percent, we performed 20 random sampling iterations for each user. Due to the high dimension of the data of dataset D4 (561 features), the number of iterations was set to 5 instead of 20 to reduce the computational time, which took approximately 20 h with 5 iterations.

**Figure 1:**
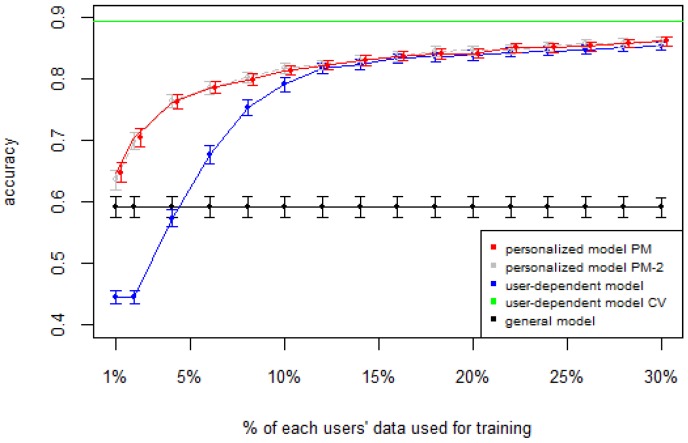
D1: Chest sensor dataset. PM: Personalized Model; CV: Cross Validated.

**Figure 2:**
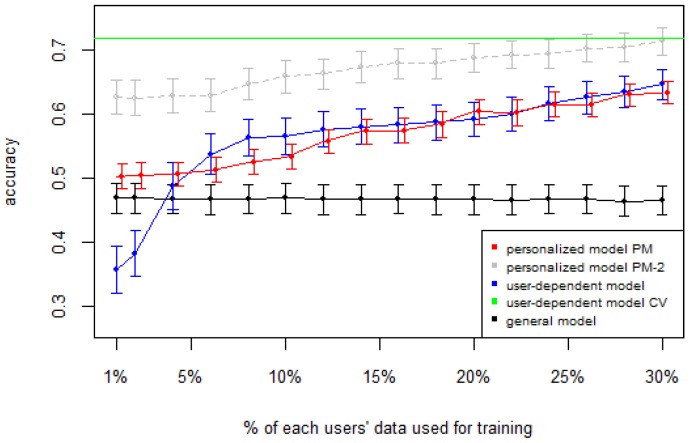
D2: Wrist sensor dataset. PM: Personalized Model; CV: Cross Validated.

**Figure 3:**
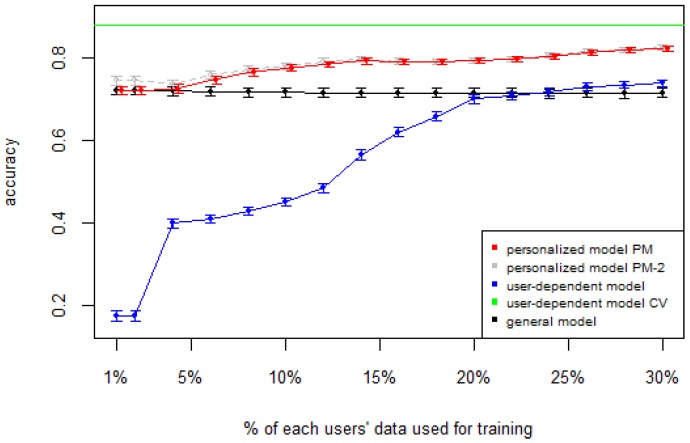
D3: WISDM dataset. PM: Personalized Model; CV: Cross Validated.

**Figure 4:**
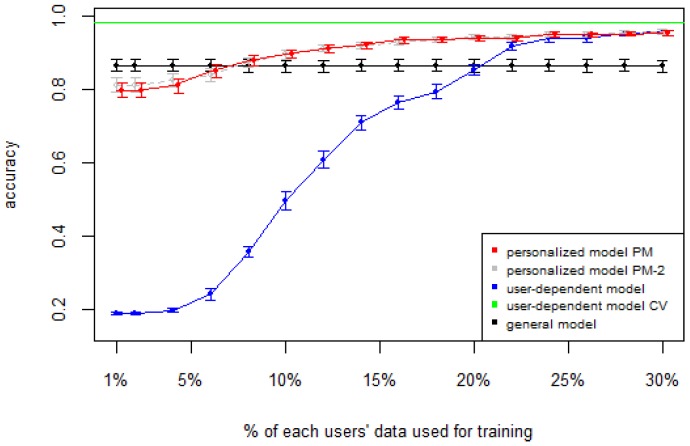
D4: Smartphone dataset. PM: Personalized Model; CV: Cross Validated.

**Figure 5:**
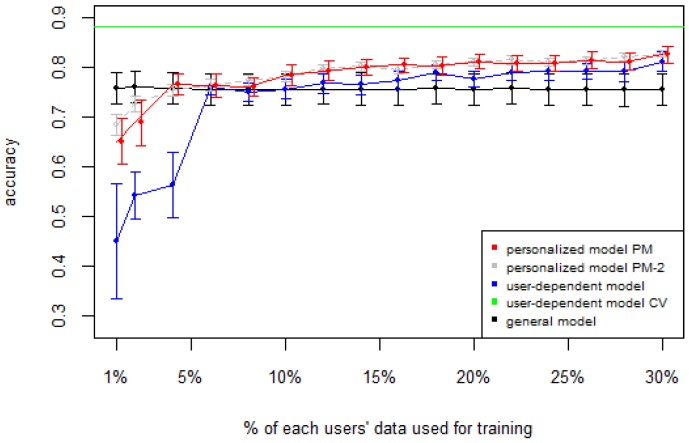
D5: Opportunity dataset. PM: Personalized Model; CV: Cross Validated.

Figure 1, Figure 2, Figure 3, Figure 4 and Figure 5 show the results of averaging the accuracy of all users for each *p* percent of data used as train set (with 95% confidence interval bars). For comparison, the figures also show the optimal case *user-dependent model CV* (Cross Validated) represented by the green line which assumes there is plenty of labeled data. This was obtained by performing 10-fold cross validation for each user independently. With this scheme, every iteration assumes 90% of the data is labeled and the other 10% is used to test the model, which is a common way of validation in the literature on Human Activity Recognition. For D1 (Figure 1), the PM clearly outperforms the other two models when the labeled data is between 1% and 10% (the curve for PM-2 will be explained soon). The GM shows a stable accuracy since it is independent of the user. For the rest of the datasets, the PM shows an overall higher accuracy except for D2 (later we will analyze why this happened). As expected, for all datasets the *user-dependent model CV* performed much better than all other models because it is built with a lot of labeled data for each specific user.

Table 2 shows the average number of labeled instances per class for each *p* percent of training data. For example, for D3 we can see how with just three labeled instances per class, the PM achieves a good classification accuracy (≈0.80).

**Figure 6:**
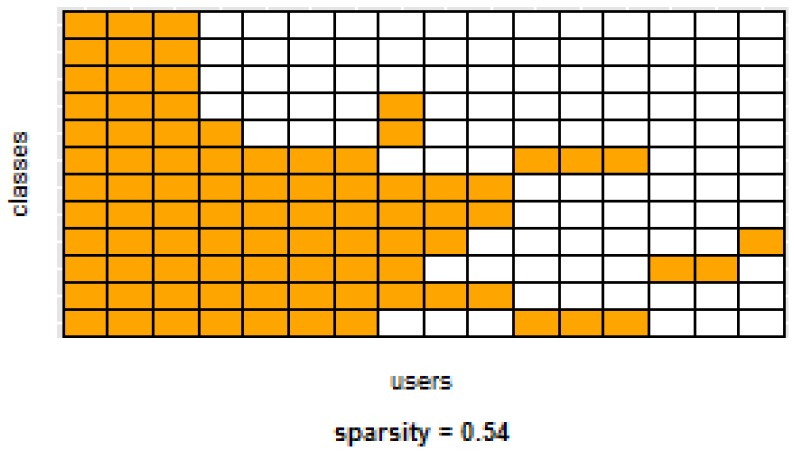
D2: Wrist sensor dataset user-class sparsity matrix.

**Figure 7:**
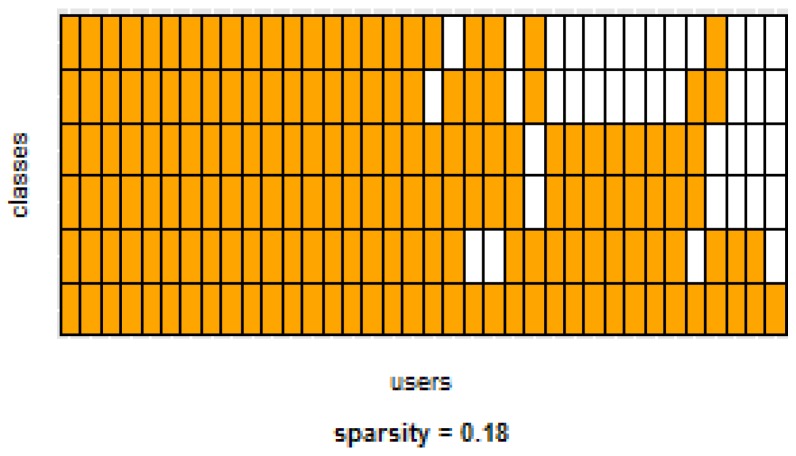
D3: WISDM dataset user-class sparsity matrix.

Table 3 and Table 4 show the difference of average overall accuracy and recall (from 1% to 30% of labeled data) between the PM and the other two models. Here we can see how the PM significantly outperforms the other two models in all datasets, except for the accuracy in D2 when comparing PM to UDM, in which case the difference is negligible. This may be due to the user-class sparsity of the dataset—*i.e.*, some users performed only a small subset of the activities. This situation will introduce noise to the PM. In the extreme case when a user has just one type of activity it would be sufficient to always predict that activity. However, the PM is trained with the entire set of possible labels from all other users, in which case the model will predict labels that are not part of that user. To confirm this, we visualized and quantified the user-class sparsity of the datasets and performed further experiments. First we computed the user-class sparsity matrices for each dataset. These matrices are generated by plotting what activities were performed by each user. A cell in the matrix is set to 1 if a user performed an activity and 0 otherwise. The sparsity index is computed as 1 minus the proportion of 1’s in the matrix. For datasets D1, D4, and D5, all users performed all activities, giving a sparsity index of 0. Figure 6 and Figure 7 show the user-class sparsity matrices of datasets D2 and D3, respectively. D2 has a sparsity index of 0.54, whereas for D3 it is 0.18. For D2, this index is very high (almost half of the entries in the matrix are 0); furthermore, the number of classes for this dataset is also high (12). From the matrix we can see that several users performed just a small number of activities (in some cases just one or two activities). One way to deal with this situation is to train the model excluding activities from other users that were not performed by the target user. Figure 1, Figure 2, Figure 3, Figure 4 and Figure 5 (gray dotted line PM-2) show the results of excluding types of activities that are not in $u_{t}$. As expected, for datasets with low or no sparsity, the results are almost the same (with small variations due to random initial *k*-means centroids). For D2 (which has a high sparsity) the accuracy significantly increased. This shows evidence that the user-class distribution of the dataset has an impact on the PM and that this can be alleviated by excluding the classes that are not relevant for a particular user.

**Figure 8:**
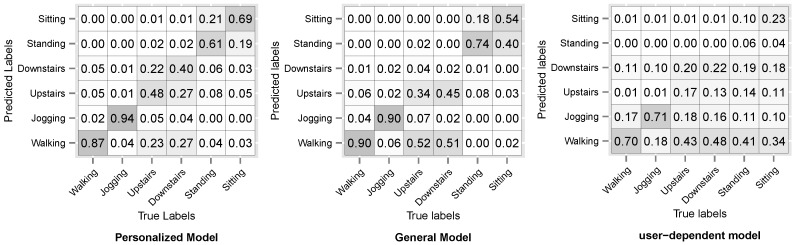
D3: WISDM sensor dataset Confusion Matrix.

**Figure 9:**
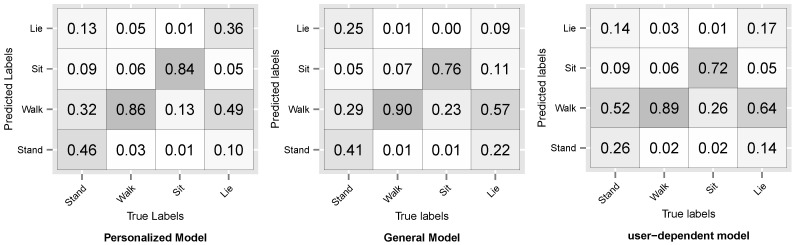
D5: Opportunity dataset Confusion Matrix.

Figure 8 and Figure 9 show the resulting confusion matrices for datasets D3 and D5. The anti-diagonal represents the recall of each individual activity. For both datasets, the recall of the general model is skewed towards the walking activity, which is also the most common. For the personalized and user-dependent model, the recall is more uniformly distributed (the anti-diagonal is more distinguishable). This behaviour was observed for the rest of the datasets.

To validate our results we used a two-tail paired *t*-test with a significance level α=0.05 to see whether or not there is a significant difference in the performance between the proposed Personalized Model and the General Model and User-Dependent Model. We also performed a Mann–Whitney U test which does not assume normality in the data. Table 5 shows the results of the statistical tests. From this table, we can see that all tests resulted in a statistically-significant performance increase, except in the case when comparing PM *vs.* UDM for dataset D2, which is the case when the sparsity was high.

For the *zero-labeling* case, the experiments were performed by selecting a random subset *R* from all the unlabeled instances $X_{u}$ from the target user. The labels of the subset *R* are predicted using a model *M* trained with data from all other users and will become the training set for the target user. The model *M* was a random forest, considered to be one with the lowest error rates across multiple classifiers [72], thus reducing the propagation of mis-classifications to the final training set. Once we have the automatically generated training set, the Personalized Model is built as usual (Procedure 1). The size of *R* is a percentage of instances *p* from $X_{u}$. We varied *p* from 0.5 to 0.8 with increments of 0.1. To account for variability, for each *p* we performed 20 iterations (except for D4, which had five iterations due to computational time) and reported the average performance. To account for dataset sparsity, the General Model and the Personalized Model were trained by removing the classes that are not part of the target user.

**Figure 10:**
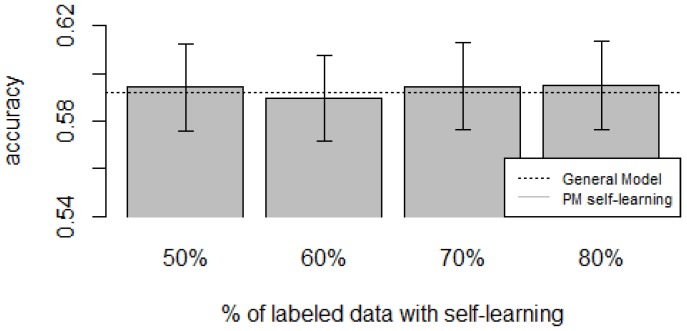
D1: Chest sensor dataset bar plot for different % of labeled data with self-learning.

**Figure 11:**
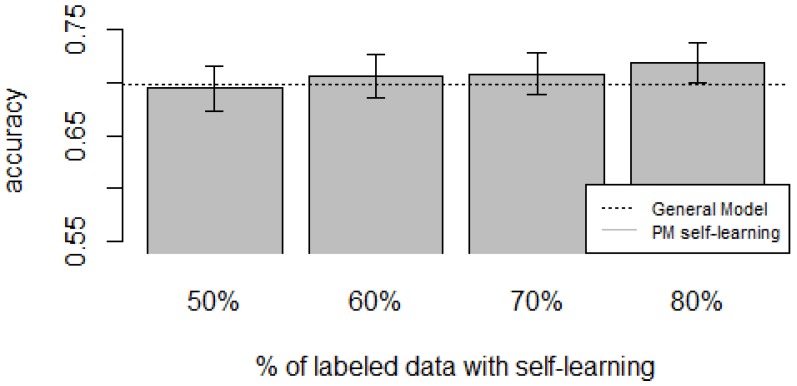
D2: Wrist sensor dataset bar plot for different % of labeled data with self-learning.

**Figure 12:**
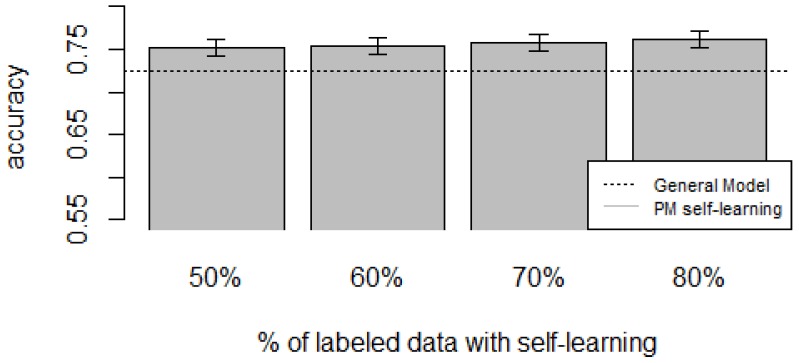
D3: WISDM dataset bar plot for different % of labeled data with self-learning.

**Figure 13:**
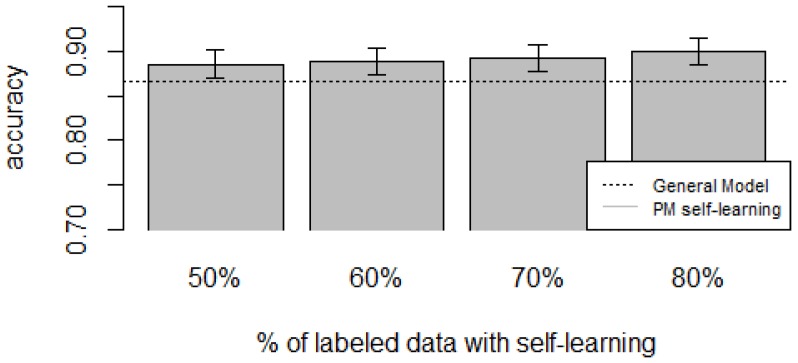
D4: Smartphone dataset bar plot for different % of labeled data with self-learning.

**Figure 14:**
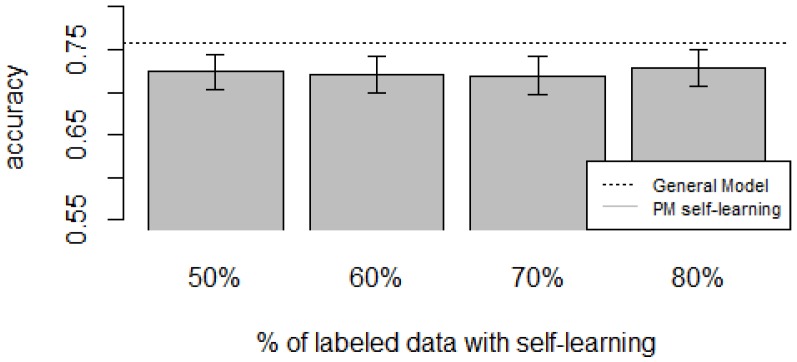
D5: Opportunity dataset bar plot for different % of labeled data with self-learning.

Figure 10, Figure 11, Figure 12, Figure 13 and Figure 14 show the obtained results. Overall, we can see that the Personalized Model when using self-training in the case of *zero-labeling* is better than the General Model (except for D5, which was worse). Furthermore, as the percentage *p* of instances used as training set increases, the accuracy does as well. Table 6, Table 7, Table 8, Table 9 and Table 10 show the same information in tabular form, with the last column being the difference in accuracy between the PM with self-learning and the General Model. From these tables, we can see that for dataset D1 the difference of the PM with respect to the GM is not considerable, and there is even a small decrease when using 60% of training data. For the rest of the datasets, the differences are more noticeable, ranging from ≈1%–3.8%. It seems that the greatest increments with respect to the GM were in datasets D3 and D4, which are the ones with more users. However, more experiments are required to validate this. With respect to datasets D1 and D2, there were no statistically significant differences (α=0.05). For D3, the difference in accuracy became statistically significant when using 60% or more training data. For D4, the increment was statistically significant when using 50% or more of the data. In the case of D5, the Personalized Model performed worse than the General Model. This may be due to the small number of users in this dataset, and thus a lack of diversity—which can limit the prediction accuracy of the self-learning phase. If the initial predictions are not accurate, these errors can propagate to the subsequent training phase, giving a deterioration in performance as a result [73]. A possible solution to this problem is to use more robust *self-labeled* algorithms [74] such as *multi-view learning* [73].

Based on our results, the proposed Personalized Model performed better than the General Model and the User-Dependent Model when there was a scarce amount of labeled data. For the case when there was no labeled data at all, the proposed approach performed better (compared to the General Model) in three (D2, D3, D4) of the five datasets, and the increment in performance was significant in two (D3, D4) of those three datasets. For D1, there was neither a noticeable increase nor decrease in performance. D5 had a statistically significant decrease in performance with respect to the General Model. Another thing to note is that for the *zero-labeling* case, much more training data was required to outperform the GM compared to the case when some labeled instances were available. For example, in dataset D3 when there was no labeling, the PM + Self-learning required 50% of automatically-generated training data to achieve an accuracy of 0.75, whereas the PM when there was just 15% of true labeled data achieved an accuracy close to 0.80. As mentioned before, this behavior is expected because the self-learning process will inevitably mis-classify some of the instances, and thus the automatically-generated ground truth will contain some errors.

## 6. Conclusions

In this work, we proposed a method based on class similarities between a collection of previous users and a specific user to build Personalized Models when labeled data for this one is scarce, thus obtaining the benefits of a “crowdsourcing” approach, where the community data is fit to the individual case. We used the small amount of labeled data from the specific user to select meaningful instances from all other users in order to reduce noise due to inter-user diversity. We evaluated the proposed method on five independent human activity datasets. The results showed a significant increase in accuracy over the General and User-Dependent Models for datasets with small sparsity. In the case of datasets with high sparsity, the performance problems were alleviated to a great extent by excluding types of activities from other users that were not performed by the target user. We also evaluated the case when there was no labeled data for the target user. In this case, we used a self-learning approach to automatically label the instances and be able to train the Personalized Model. Our experiments showed that there was an increase in accuracy with respect to the General Model in three of the five datasets and on two of these datasets this increase was statistically significant. For dataset five, however, the Personalized Model experienced a decrease in performance. The self-learning process inevitably introduces some errors in the generation of the training set. Methods that can provide some guard against this type of error will be explored for future work.

In this work, we assumed that the users collected the data using the same type of device. An interesting future direction would be to also take into account the heterogeneity of the hardware. For our experiments, we also assumed that all the possible types of activities are known; however, in real situations, the user might perform activities that do not correspond to any predefined ones (unknown activities), but the classifier will try to assign them a label anyway. As noted by Reyes *et al.* [75] (who also proposed a method to deal with this problem), this can introduce errors in the system. Dealing with these unknown activities is also worth considering if the system is to be deployed in production environments. Another future direction is to carry this type of crowdsourcing-based training on long-term/complex activities [76], like commuting, shopping, cooking, dining, *etc*.

## Figures and Tables

**Table 1:** Datasets summary.

Abbreviation	Name	# Subjects	# Considered Classes	# Instances
D1	Chest Sensor	15	4	8506
D2	Wrist Sensor	16	12	2807
D3	WISDM	36	6	5418
D4	Smartphone	21	6	7352
D5	Opportunity	4	4	1855

**Table 2:** Average number of labeled instances per class for each dataset.

	1%	5%	10%	15%	20%
D1	1	7	14	21	28
D2	1	1	2	3	3
D3	1	2	3	4	5
D4	1	3	6	9	12
D5	1	5	11	17	23

**Table 3:** Difference of average overall accuracy (from 1% to 30% of labeled data) between the Personalized Model and the other two models. PM: Personalized model; GM: General model; UDM: User-dependent model.

	PM-GM	PM-UDM
D1	22.4%	4.7%
D2	10.2%	0.001%
D3	6.3%	22.3%
D4	4.0%	25.5%
D5	2.9%	4.5%

**Table 4:** Difference of average overall recall (from 1% to 30% of labeled data) between the Personalized Model and the other two models. PM: Personalized model; GM: General model; UDM: User-dependent model.

	PM-GM	PM-UDM
D1	18.2%	7.9%
D2	9.5%	16.6%
D3	7.2%	34.1%
D4	4.3%	28.0%
D5	6.1%	11.9%

**Table 5:** Results of the statistical tests. PM: Personalized model; GM: General model; UDM: User-dependent model.

	PM/GM		PM/UDM	
	*t*-Test	Mann-Withney	*t*-Test	Mann-Withney
D1: Chest sensor	p<<0.01	p<<0.01	p<<0.01	p<<0.01
D2: Wrist sensor	p<<0.01	p<<0.01	p>>0.05	p>>0.05
D3: Wisdm	p<<0.01	p<<0.01	p<<0.01	p<<0.01
D4: Smartphone	p<<0.01	p<<0.01	p<<0.01	p<<0.01
D5: Opportunity	p<<0.01	p<<0.01	p<<0.01	p<<0.01

**Table 6:** D1: Chest sensor dataset accuracies for varying % of labeled instances with self-learning (PM + self-learning) and the General Model (GM).

% Labeled Instances	PM + Self-Learning	GM	Difference
50%	0.5941825	0.5919693	0.002213172
60%	0.5894306	0.5919693	−0.002538676
70%	0.5945105	0.5919693	0.002541170
80%	0.5949319	0.5919693	0.002962601

**Table 7:** D2: Wrist sensor dataset accuracies for varying % of labeled instances with self-learning (PM + self-learning) and the General Model (GM).

% Labeled Instances	PM + Self-Learning	GM	Difference
50%	0.6949760	0.6976796	−0.002703561
60%	0.7060070	0.6976796	0.008327441
70%	0.7081631	0.6976796	0.010483591
80%	0.7189719	0.6976796	0.021292339

**Table 8:** D3: WISDM dataset accuracies for varying % of labeled instances with self-learning (PM + self-learning) and the General Model (GM).

% Labeled Instances	PM + Self-Learning	GM	Difference
50%	0.7513043	0.7249541	0.02635025
60%	0.7530312	0.7249541	• 0.02807717
70%	0.7580999	0.7249541	• 0.03314587
80%	0.7617153	0.7249541	• 0.03676125

• statistically significant difference.

**Table 9:** D4: Smartphone dataset accuracies for varying % of labeled instances with self-learning (PM + self-learning) and the General Model (GM).

% Labeled Instances	PM + Self-Learning	GM	Difference
50%	0.8850233	0.8655395	• 0.01948381
60%	0.8879735	0.8655395	• 0.02243403
70%	0.8925108	0.8655395	• 0.02697124
80%	0.8992250	0.8655395	• 0.03368545

• statistically significant difference.

**Table 10:** D5: Opportunity dataset accuracies for varying % of labeled instances with self-learning (PM + self-learning) and the General Model (GM).

% Labeled Instances	PM + Self-Learning	GM	Difference
50%	0.7238851	0.7575434	• −0.03365834
60%	0.7207967	0.7575434	• −0.03674673
70%	0.7193240	0.7575434	• −0.03821944
80%	0.7282631	0.7575434	• −0.02928034

• statistically significant difference.
